# Supplementary material for: Molecular Epidemiology of SARS-CoV-2 Omicron Sub-Lineages Isolated from Turkish Patients Infected with COVID-19
Source: Viruses. 2023 Apr 27;15(5):1066. doi: 10.3390/v15051066 (PMC10223230; doi:10.3390/v15051066)
Supplement: Supplementary file 1 [file viruses-15-01066-s001.zip › viruses-2323881-supplementary.pdf]

viruses

Table 1. Distribution of SARS-CoV-2 Omicron sublineages isolated in the Turkish population between December 2021 to February 2023.

SARS-CoV-2 Omicron sub-lineage	Descendent lineage	GISAID sequence n, %
**B.1**		**354 (1.68)**
	B.1	10 (2.82)
	B.1.1	6 (1.69)
	B.1.1.529	331 (93.5)
	B.1.617.2	7 (1.97)
**BA.1**		**7293 (34.7)**
	BA.1	6010 (82.40)
	BA.1.1	917 (12.5)
	BA.1.1.1	103 (1.41)
	BA.1.1.11	1 (0.01)
	BA.1.1.14	5 (0.06)
	BA.1.1.15	2 (0.02)
	BA.1.1.16	1 (0.01)
	BA.1.1.18	2 (0.02)
	BA.1.10	2 (0.02)
	BA.1.12	1 (0.01)
	BA.1.13	2 (0.02)
	BA.1.14	20 (0.27)
	BA.1.14.2	2 (0.02)
	BA.1.15	42 (0.57)
	BA.1.15.1	13 (0.17)
	BA.1.16	2 (0.02)
	BA.1.17	72 (0.98)
	BA.1.17.2	56 (0.76)
	BA.1.18	27 (0.37)
	BA.1.19	2 (0.02)
	BA.1.20	1 (0.01)
	BA.1.4	1 (0.01)
	BA.1.7	1 (0.01)
	BA.1.9	8 (0.10)
**BA.2**		**6463 (30.7)**
	BA.2	5588 (86.46)
	BA.2.1	15 (0.23)
	BA.2.10	34 (0.52)
	BA.2.10.1	10 (0.15)
	BA.2.12	7 (0.10)
	BA.2.12.1	69 (1.06)
	BA.2.13	7 (0.10)
	BA.2.14	3 (0.04)
	BA.2.17	2 (0.03)
	BA.2.18	4 (0.06)
	BA.2.22	2 (0.03)

	BA.2.23	16 (0.24)
	BA.2.24	1 (0.01)
	BA.2.25	1 (0.01)
	BA.2.27	4 (0.06)
	BA.2.29	1 (0.01)
	BA.2.3	153 (2.36)
	BA.2.3.15	2 (0.03)
	BA.2.3.20	1 (0.01)
	BA.2.3.4	1 (0.01)
	BA.2.31	4 (0.06)
	BA.2.31.1	3 (0.04)
	BA.2.32	4 (0.06)
	BA.2.33	1 (0.01)
	BA.2.35	2 (0.03)
	BA.2.36	10 (0.15)
	BA.2.37	4 (0.06)
	BA.2.38	1 (0.01)
	BA.2.40.1	3 (0.04)
	BA.2.41	9 (0.13)
	BA.2.43	2 (0.03)
	BA.2.48	1 (0.01)
	BA.2.5	70 (1.08)
	BA.2.52	4 (0.06)
	BA.2.56	12 (0.18)
	BA.2.57	1 (0.01)
	BA.2.58	7 (0.10)
	BA.2.6	1 (0.01)
	BA.2.62	2 (0.03)
	BA.2.63	9 (0.13)
	BA.2.65	7 (0.10)
	BA.2.7	5 (0.07)
	BA.2.71	2 (0.03)
	BA.2.72	2 (0.03)
	BA.2.73	1 (0.01)
	BA.2.75	121 (1.87)
	BA.2.75.2	2 (0.03)
	BA.2.75.5	1 (0.01)
	BA.2.75.7	1 (0.01)
	BA.2.76	5 (0.07)
	BA.2.79	3 (0.04)
	BA.2.8	1 (0.01)
	BA.2.9	232 (3.58)
	BA.2.9.2	1 (0.01)
	BA.2.9.3	2 (0.03)
	BA.2.9.5	4 (0.06)
	BA.2.9.6	2 (0.03)
**BA.4**		**93 (0.44)**
	BA.4	46 (49.46)
	BA.4.1	21 (22.5)
	BA.4.1.1	6 (6.45)
	BA.4.1.3	2 (2.15)

	BA.4.1.6	1 (1.07)
	BA.4.4	1 (1.07)
	BA.4.6	6 (6.45)
	BA.4.6.3	2 (2.15)
	BA.4.6.5	3 (3.22)
	BA.4.7	1 (1.07)
	BA.4.8	4 (4.30)
**BA.5**		**4953 (23.5)**
	BA.5	163 (3.29)
	BA.5.1	435 (8.78)
	BA.5.1.1	3 (0.06)
	BA.5.1.10	6 (0.12)
	BA.5.1.12	1 (0.02)
	BA.5.1.17	2 (0.04)
	BA.5.1.18	1 (0.02)
	BA.5.1.2	8 (0.16)
	BA.5.1.22	58 (1.17)
	BA.5.1.23	25 (0.50)
	BA.5.1.24	18 (0.36)
	BA.5.1.26	9 (0.18)
	BA.5.1.3	14 (0.28)
	BA.5.1.30	6 (0.12)
	BA.5.1.4	8 (0.16)
	BA.5.1.5	5 (0.10)
	BA.5.10.1	1 (0.02)
	BA.5.2	2095 (42.2)
	BA.5.2.1	1172 (23.6)
	BA.5.2.10	5 (0.10)
	BA.5.2.12	5 (0.10)
	BA.5.2.13	3 (0.06)
	BA.5.2.16	76 (1.53)
	BA.5.2.18	13 (0.26)
	BA.5.2.19	5 (0.10)
	BA.5.2.2	1 (0.02)
	BA.5.2.20	109 (2.20)
	BA.5.2.21	21 (0.42)
	BA.5.2.22	12 (0.24)
	BA.5.2.24	15 (0.30)
	BA.5.2.26	5 (0.10)
	BA.5.2.27	21 (0.42)
	BA.5.2.28	39 (0.78)
	BA.5.2.3	7 (0.14)
	BA.5.2.31	14 (0.28)
	BA.5.2.32	4 (0.08)
	BA.5.2.33	2 (0.04)
	BA.5.2.34	28 (0.56)
	BA.5.2.35	1 (0.02)
	BA.5.2.36	6 (0.12)
	BA.5.2.38	1 (0.02)
	BA.5.2.4	1 (0.02)
	BA.5.2.44	2 (0.04)

	BA.5.2.6	228 (4.60)
	BA.5.2.7	31 (0.62)
	BA.5.2.8	8 (0.16)
	BA.5.2.9	14 (0.28)
	BA.5.3	29 (0.58)
	BA.5.3.1	58 (1.17)
	BA.5.3.2	4 (0.08)
	BA.5.3.3	6 (0.12)
	BA.5.5	6 (0.12)
	BA.5.6	108 (2.18)
	BA.5.8	3 (0.06)
	BA.5.9	32 (0.64)
**BE.1**		**285 (1.35)**
	BE.1	130 (45.61)
	BE.1.1	132 (46.3)
	BE.1.1.1	3 (1.05)
	BE.1.1.2	18 (6.31)
	BE.1.2	2 (0.70)
**BF.1**		**21 (0.10)**
**BF.28**		**41 (0.19)**
**BF.5**		**138 (0.65)**
**BF.6**		**20 (0.09)**
**BF.7**		**49 (0.23)**
	BF.7	36 (73,47)
	BF.7.1	1 (2.04)
	BF.7.4	7 (14.2)
	BF.7.5	2 (4.08)
	BF.7.6	2 (4.08)
	BF.7.8	1 (2.04)
**BM.4.1**		**27 (0.12)**
**BN.1**		**62 (0.29)**
	BN.1.2	2 (3.22)
	BN.1.3	20 (32.2)
	BN.1.3.1	9 (14.5)
	BN.1.4	15 (24.1)
	BN.1.5	2 (3.22)
	BN.1.7	1 (1.61)
	BN.1.9	12 (19.3)
**BN.3.1**		**25 (0.11)**
**BQ.1**		**834 (3.97)**
	BQ.1	442 (52.99)
	BQ.1.1	100 (11.9)
	BQ.1.1.1	1 (0.11)
	BQ.1.1.10	2 (0.23)
	BQ.1.1.11	1 (0.11)
	BQ.1.1.13	2 (0.23)
	BQ.1.1.15	1 (0.11)
	BQ.1.1.17	11 (1.31)
	BQ.1.1.18	15 (1.79)
	BQ.1.1.2	1 (0.11)
	BQ.1.1.22	12 (1.43)

	BQ.1.1.23	1 (0.11)
	BQ.1.1.24	2 (0.23)
	BQ.1.1.26	2 (0.23)
	BQ.1.1.28	4 (0.47)
	BQ.1.1.29	2 (0.23)
	BQ.1.1.3	8 (0.95)
	BQ.1.1.31	1 (0.11)
	BQ.1.1.4	10 (1.19)
	BQ.1.1.5	1 (0.11)
	BQ.1.1.7	4 (0.47)
	BQ.1.1.8	3 (0.35)
	BQ.1.10	10 (1.19)
	BQ.1.10.1	2 (0.23)
	BQ.1.11	3 (0.35)
	BQ.1.12	45 (5.39)
	BQ.1.13	2 (0.23)
	BQ.1.14	7 (0.83)
	BQ.1.16	2 (0.23)
	BQ.1.18	3 (0.35)
	BQ.1.19	2 (0.23)
	BQ.1.2	47 (5.63)
	BQ.1.20	1 (0.11)
	BQ.1.22	1 (0.11)
	BQ.1.23	56 (6.71)
	BQ.1.25	28 (3.35)
	BQ.1.3	76 (9.11)
	BQ.1.5	6 (0.71)
	BQ.1.6	6 (0.71)
	BQ.1.8	1 (0.11)
**CK.1**		**27 (0.12)**
**CL.1**		**34 (0.16)**
**XBB.1**		**70 (0.33)**
	XBB.1	60 (85.71)
	XBB.1.2	4 (5.71)
	XBB.1.9	6 (8.57)
**Others***		**191 (0.91)**
**Unassigned****		**9 (0.04)**
TOTAL		20.959 (100)

* **Others** Sub lineages and descendent variants that were determined at low rates: AY.1 n=5(0.023%), AY.100 n=3(0.014%), AY.102 n=1(0.004%), AY.121 n=6(0.028%), AY.122 n=2(0.009%), AY.126 n=1(0.004%), AY.33 n=1(0.004%), AY.42 n=1(0.004%), BA.3 n=6(0.028%), BE.3 n=1(0.004%), BE.4 n=3(0.014%), BF.10 n=11(0.052%), BF.11 n=11(0.052%), BF.11.1 n=2(0.009%), BF.11.2 n=1(0.004%), BF.14 n=13(0.061%), BF.15 n=4(0.019%), BF.18 n=2(0.009%), BF.2 n=2(0.009%), BF.21 n=1(0.004%), BF.24 n=1(0.004%), BF.26 n=3(0.014%), BF.27 n=1(0.004%), BF.31 n=1(0.004%), BF.4 n=5(0.023%), BJ.1 n=1(0.004%), BL.1 n=1(0.004%), BM.1.1 n=1(0.004%), BM.1.1.1 n=4(0.019%), BM.1.1.3 n=2(0.009%), BM.1.1.4 n=1(0.004%), BM.2 n=1(0.004%), BN.2 n=2(0.009%), BT.2 n=1(0.004%), BU.1 n=2(0.009%), BV.1 n=13(0.061%), BY.1 n=3(0.014%), BY.1.2 n=4(0.019%), CH.1.1 n=4(0.019%), CH.1.1.1 n=2(0.009%), CH.1.1.3 n=1(0.004%), CK.2 n=1(0.004%), CK.2.1.1 n=1(0.004%), CK.3 n=1(0.004%), CM.2 n=1(0.004%), CN.1 n=4(0.019%), CP.1 n=1(0.004%), CP.1.1 n=1(0.004%), CP.2 n=14(0.066%), CP.3 n=4(0.019%), CP.4 n=3(0.014%), CQ.1 n=1(0.004%), CR.1 n=4(0.019%), DE.1 n=2(0.009%), DE.2 n=3(0.014%), DM.1 n=3(0.014%), DR.1 n=1(0.004%), XAZ n=3(0.014%), XBB.2 n=2(0.009%), XBB.2.2

n=1(0.004%), XBB.3 n=1(0.004%), XBC.1 n=1(0.004%), XBD n=1(0.004%), XBF n=1(0.004%), XBK n=4(0.019%), XM n=1(0.004%).

**: Amino acid substitutions that were not defined as sub-variants.

Note: Omicron subvariants/descendent variants were given in alphabetic order in the table.
